# Replacing meat with alternative plant-based products (RE-MAP): a randomized controlled trial of a multicomponent behavioral intervention to reduce meat consumption

**DOI:** 10.1093/ajcn/nqab414

**Published:** 2021-12-27

**Authors:** Filippo Bianchi, Cristina Stewart, Nerys M Astbury, Brian Cook, Paul Aveyard, Susan A Jebb

**Affiliations:** Nuffield Department of Primary Care Health Sciences, University of Oxford, Oxford, UK; Nuffield Department of Primary Care Health Sciences, University of Oxford, Oxford, UK; Nuffield Department of Primary Care Health Sciences, University of Oxford, Oxford, UK; National Institute for Health Research, Oxford Biomedical Research Centre, Oxford University Hospitals, National Health Service Foundation Trust, Oxford, UK; Nuffield Department of Primary Care Health Sciences, University of Oxford, Oxford, UK; Nuffield Department of Primary Care Health Sciences, University of Oxford, Oxford, UK; National Institute for Health Research, Oxford Biomedical Research Centre, Oxford University Hospitals, National Health Service Foundation Trust, Oxford, UK; Nuffield Department of Primary Care Health Sciences, University of Oxford, Oxford, UK; National Institute for Health Research, Oxford Biomedical Research Centre, Oxford University Hospitals, National Health Service Foundation Trust, Oxford, UK

**Keywords:** meat substitutes, vegetarian, meat, food choice motives, food neophobia, consumer attitudes, consumer acceptance, sustainability

## Abstract

**Background:**

Reducing meat consumption could protect the environment and human health.

**Objectives:**

We tested the impact of a behavioral intervention to reduce meat consumption.

**Methods:**

Adult volunteers who regularly consumed meat were recruited from the general public and randomized 1:1 to an intervention or control condition. The intervention comprised free meat substitutes for 4 weeks, information about the benefits of eating less meat, success stories, and recipes. The control group received no intervention or advice on dietary change. The primary outcome was daily meat consumption after 4 weeks, assessed by a 7-day food diary, and repeated after 8 weeks as a secondary outcome. Other secondary and exploratory outcomes included the consumption of meat substitutes, cardiovascular risk factors, psychosocial variables related to meat consumption, and the nutritional composition of the diet. We also estimated the intervention's environmental impact. We evaluated the intervention using generalized linear mixed-effects models.

**Results:**

Between June 2018 and October 2019, 115 participants were randomized. The baseline meat consumption values were 134 g/d in the control group and 130 g/d in the intervention group. Relative to the control condition, the intervention reduced meat consumption at 4 weeks by 63 g/d (95% CI: 44–82; *P* < 0.0001; *n* = 114) and at 8 weeks by 39 g/d (95% CI: 16–62; *P* = 0.0009; *n* = 113), adjusting for sex and baseline consumption. The intervention significantly increased the consumption of meat substitutes without changing the intakes of other principal food groups. The intervention increased intentions, positive attitudes, perceived control, and subjective norms of eating a low-meat diet and using meat substitutes, and decreased attachment to meat. At 8 weeks, 55% of intervention recipients identified as meat eaters, compared to 89% of participants in the control group.

**Conclusions:**

A behavioral program involving free meat substitutes can reduce meat intake and change psychosocial constructs consistent with a sustained reduction in meat intake.

See corresponding editorial on page 1263.

## Introduction

Reducing meat consumption could help to protect the natural environment, and lower intake of red and processed meat is linked with reduced risks of numerous chronic conditions (1). Plant-based meat substitutes, such as those made of textured vegetable protein or mycoprotein, could enable meat eaters to replace meat with plant-based products without changing their wider dietary habits (2). However, despite the growing range and availability of meat substitutes, their consumption in developed countries remains low (3–6). This might partly be because many people perceive meat substitutes to be unfamiliar products and do not consider them to be acceptable alternatives to meat (7, 8). Repeated exposure to free meat substitutes could increase people's familiarity with and liking for these products. This could occur through a process of mere exposure: that is, people's tendency to develop a liking towards things simply as they become more familiar with them (9–11). We developed a complex behavioral intervention centered around the provision of free meat substitutes for 4 weeks, as well as information on the benefits of eating less meat, success stories, and recipes [described in full elsewhere (8)], and evaluated its effect in a randomized controlled trial (RCT). The aim of the study was to test whether participants can reduce their meat consumption when meat-free replacements are provided free for 4 weeks, and what happens when the provision of meat-free substitutes ends. The objectives include the impact of the intervention on recipients’ meat consumption, wider dietary intakes, psychosocial variables related to the intake of meat and meat substitutes (e.g., attitudes towards eating a low-meat diet or using meat substitutes), and cardiovascular risk factors. We also estimated the effect of the intervention on the environmental impact of the diet attributable to food production.

## Methods

### Study design

The full study design is described elsewhere (12). Briefly, this was a parallel, 2-arm, individually randomized controlled trial of a multicomponent behavioral intervention to reduce meat consumption. The study was conducted in Oxford, UK, among participants from adult-only households recruited from the community through advertisements. People were eligible if they ate meat at least 5 times per week and did not eat meat substitutes regularly. The study included 4 visits: at the enrolment appointment, we collected written informed consent and trained volunteers to record a 7-day food diary app using MyFitnessPal, which has been used in previous studies and validated against paper 7-day food diaries (13, 14).

Participants who completed their food diary to a good standard (defined as 5 or more days with diary entries of 1000+ kcal/d) were invited to a baseline visit and were randomized in a 1:1 ratio to the intervention or the control condition. The allocation sequence was generated by an independent statistician using random permuted blocks and was stratified by sex. Group allocation was revealed to the researcher once eligibility had been confirmed and baseline data collection was completed, thus ensuring full allocation concealment during the baseline data collection. Neither the participants nor the researcher conducting the appointments were blind to group allocation, but the researcher coding the food diaries and entering the data in a bespoke online database was blinded.

Participants allocated to the intervention group received the behavioral intervention for 4 weeks, while those in the control condition received no support to reduce their meat consumption and no further dietary advice. All participants were invited to attend a 4-week follow-up at the end of the intervention period and an 8-week follow-up 4 weeks after the intervention had finished.

All participants were asked to keep a 7-day food diary using MyFitnessPal leading up to each appointment. At each follow-up visit, body weight and body composition were measured using an electronic scale (Tanita), blood pressure was measured, and a finger-prick capillary blood sample was collected to measure blood lipid profiles using a point-of-care device (Alere Cholestech LDX). Participants were asked to complete questionnaires assessing psychosocial outcomes. Serious adverse events were to be tracked as per Good Clinical Practice from enrolment until after the end of the 8-week follow-up; however, there were no serious adverse events. The contact times were similar for both groups, with the exception of an additional phone call to the intervention group after 2 weeks to arrange further food deliveries. This was entirely transactional and did not include any additional support.

To promote the completion of food diaries and attendance to study visits, we sent reminder text messages and provided financial compensation (12). The trial was approved by the Medical Sciences Interdivisional Research Ethics Committee of the University of Oxford (ref: R54329/RE001) and registered prospectively (12).

### Intervention and control

The full intervention has been described in full previously (12). Briefly, the intervention was developed following the Behavior Change Wheel (15) and involved providing 4 components:

1. free meat substitutes for the household for 4 weeks;
2. information leaflets about the health and environmental benefits of eating less meat;
3. recipes; and
4. success stories in the form of vignettes of people who reduced their meat intake.

Participants selected the meat-free substitute foods from a catalogue containing the full range of products available in a major UK grocery store at the time of the trial. This included mycoprotein meat alternatives and vegetable- and pulse-based meat substitutes. They selected the quantities for their household for 2 weeks, with a follow-up delivery for the second 2 weeks of the intervention. The range of products offered were widely available to purchase from local grocery stores.

The intervention content was developed collaboratively with members of the public. The control condition involved no intervention or additional dietary advice to participants.

### Outcomes

The primary outcome was the change in meat consumed from baseline (T0) to 4 weeks of follow-up (T1). Meals containing meat were identified from the food diaries, and meat consumption in grams per day was estimated by disaggregating meat and meat-containing products recorded on participants’ 7-day food diaries. The disaggregation procedure involved estimating the weight of the overall product; estimating the proportion of the product that was meat; converting the weight of uncooked meat to the weight of cooked meat, if appropriate; and then categorizing meat into the different sub-types.

The secondary outcomes were the change in meat consumption at 8 weeks and changes at 4 and 8 weeks in psychosocial variables related to meat consumption and eating identities. Prespecified exploratory outcomes included changes in the consumption frequencies of main food groups, psychosocial variables related to using meat substitutes, the nutritional composition of the diet, selected biomarkers of cardiovascular risk (weight, body fat, blood pressure, and blood lipid profile), and change in the desire for meat substitutes to be similar to meat. We also estimated the impact of the intervention on the greenhouse gas (GHG) emissions and land use from participants’ diets using the following method. First, we estimated participants’ consumption of the different food groups in grams per week. For meat products, we used the data from disaggregated food diaries; for other food groups, we multiplied the self-reported “number of weekly meals” containing a specific food group by a standard portion size for said food. The amount of CO_2_-eq emitted to produce 1 gram of each food group was derived from the database by Poore and Nemecek (16). Since this database did not include any information on the environmental impact of mycoprotein (or a reasonable proxy thereof), we used the results of a separate lifecycle analysis to estimate the GHG emissions associated with producing mycoprotein (17). As it was impossible to make reasonable assumptions about the environmental footprint of “snacks” without more granular information about this food group, snacks were excluded from this analysis. The land use (in m^2^) associated with participants’ weekly diets was estimated by applying the same methodology, data sources, and assumptions as those employed to estimate GHG emissions, except that we multiplied participants’ consumption of food groups (in grams/week) times the m^2^ of land used to produce 1 gram of each food included in the analysis.

### Sample size and statistical analysis

A sample of 100 volunteers allowed detection of a medium effect size (d) of 0.6 with 84% power and an alpha of 0.05 (12). Following the intention-to-treat approach, participants were analyzed according to the study group they were allocated to. The prespecified main analyses used generalized linear mixed-effects models based on all available data points. A sensitivity analysis was conducted using baseline observation carried forward (BOCF), an imputation technique commonly used in clinical trials in which the baseline value of the outcome of interest is used to impute any missing values of participants who dropped out from the study. For the primary analysis, the models included randomized group, visit, the interaction between visit and randomized group, baseline meat consumption, and sex as fixed effects, while the intercept and slope were included as random effects to account for repeated measures on the same participant. Analogous linear or logistic models were used for other outcomes. All analyses were conducted in Stata 16 (version 14.1, StataCorp LLC, College Station, TX, USA).

### Deviations from protocol

We changed our protocol to adjust our analyses for sex, as that was a stratification variable in the randomization (18). We conducted sensitivity analyses to assess the impact of this deviation on the results, and found little difference between the unadjusted and adjusted analyses.

## Results

**FIGURE 1:**
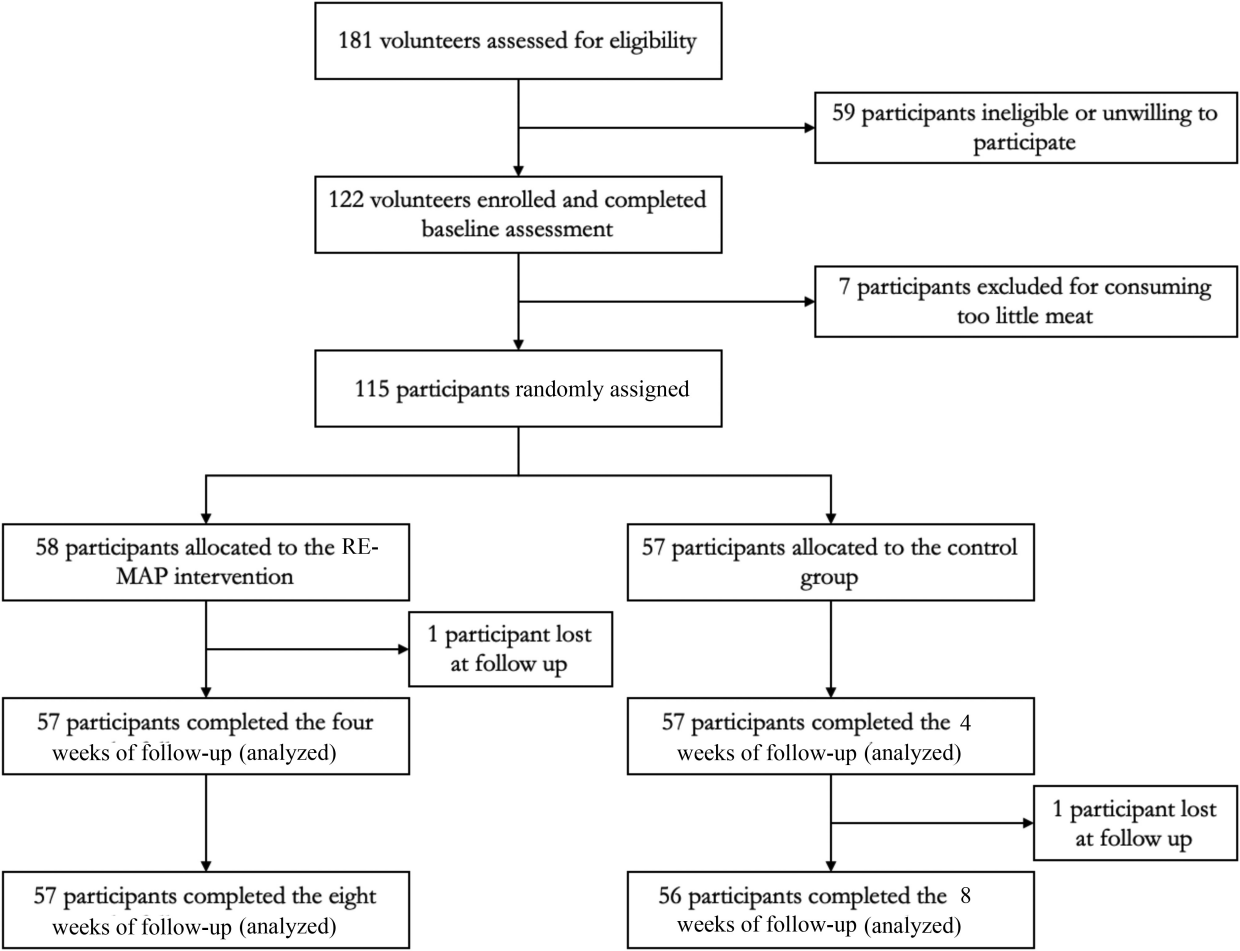
Flowchart depicting the passage of subjects through the study. Using an intention-to-treat approach, participants were analyzed according to the study group they were allocated to.

Participants were recruited between 29 June 2018 and 10 October 2019. In total, 181 volunteers contacted the research team, 122 were initially eligible and consented, but 7 were excluded before randomization because their meat consumption was below the eligibility criterion. Of 115 randomized participants, 58 were allocated to the intervention group and 57 to the control group. Two participants were lost to follow-up, 1 in each study group (**Figure 1**).

Participants were mostly aged between their mid-20s to mid-40s, two-thirds were women, the majority had a college degree, and about half lived alone (**Table 1**). Baseline meat consumption was 134 g/d in the control and 130 g/d in the intervention group. Relative to the control group, the intervention reduced meat consumption by 63 g/d (95% CI: 44–82; *P* < 0.0001) at 4 weeks and 39 g/d (95% CI: 16–62; *P* = 0.0009) at 8 weeks (**Table 2**). In the BOCF analysis, relative to the control group, the intervention reduced meat consumption by 61 g/d (95% CI: 42–80; *P* < 0.0001) at 4 weeks and 38 g/d (95% CI: 15–66; *P* = 0.0011) at 8 weeks.

**TABLE 1:** Baseline characteristics of enrolled participants (*n* = 115)

	Control group (*n* = 57)	Intervention group (*n* = 58)
Age, years	37 (12)	33 (11)
Gender, *n* (%)		
Female	37 (65)	38 (66)
Male	19 (33)	19 (33)
Other/prefer not to say	1 (2)	1 (2)
Ethnic origin, *n* (%)		
White	45 (79)	50 (86)
Chinese	2 (4)	4 (7)
Black Caribbean/African	1 (2)	1 (2)
Other/prefer not to say	9 (16)	3 (5)
Weight, kg	73.3 (16.2)	74.2 (26.6)
BMI, kg/m^2^	25.2 (5)	25.5 (5.6)
Body fat percentage, %	27 (10)	27 (11)
Total cholesterol, mmol/L	4.5 (0.9)	4.5 (0.9)
HDL cholesterol, mmol/L	1.3 (0.4)	1.4 (0.4)
Non-HDL cholesterol, mmol/L	3.1 (0.9)	3.1 (0.9)
LDL cholesterol, mmol/L	2.6 (0.8)	2.5 (0.9)
Triglycerides, mmol/L	1.3 (0.7)	1.3 (0.8)
Systolic blood pressure, mmHg	117 (14)	117 (12)
Diastolic blood pressure, mmHg	79 (9)	79 (8)
Total meat consumption, g/day	134 (72)	130 (79)

Data are presented as mean (SD), unless otherwise stated. Participants were drawn from adult-only households.

**TABLE 2:** Results^1^

	4 weeks		8 weeks		Adjusted mean difference from baseline (95% CI)^2^			
	Intervention (*n* = 57)^3^	Control (*n* = 57)^4^	Intervention (*n* = 56)^5^	Control (*n* = 56)^6^	4 weeks (*n* = 114)^7^	*P* value	8 weeks (*n* = 112)^8^	*P* value
Total meat consumption,^9^ g/day	51 (49)	116 (63)	81 (78)	122 (70)	−63 (−82 to −44)	<0.0001	−39 (−62 to −16)	0.0009
Weight,^10^ kg	73.1 (19.8)	73.3 (16.2)	72.7 (20)	73.5 (16.2)	−0.5 (−0.9 to −0.2)	0.0037	−0.6 (−1.2 to −0.1)	0.0266
BMI, kg/m^2^	25.2 (5.4)	25.2 (5.1)	25.1 (5.5)	25.2 (5)	−0.2 (−0.3 to −0.1)	0.004	−0.2 (−0.4 to 0)	0.0307
Body fat percentage, %	26.9 (10.8)	27.2 (9.8)	26.4 (11)	27.4 (9.7)	−0.2 (−0.7 to 0.3)	0.4245	−0.8 (−1.7 to 0.1)	0.0887
Total cholesterol, mmol/L	4.5 (1)	4.5 (0.8)	4.6 (1)	4.6 (0.8)	−0.1 (−0.3 to 0.1)	0.2125	0 (−0.2 to 0.1)	0.6151
HDL cholesterol, mmol/L	1.4 (0.4)	1.4 (0.4)	1.4 (0.4)	1.4 (0.4)	0 (−0.1 to 0)	0.218	0 (−0.1 to 0)	0.3103
Non-HDL cholesterol, mmol/L	3.1 (1)	3.2 (0.9)	3.2 (1)	3.2 (0.9)	0 (−0.2 to 0.1)	0.5648	0 (−0.2 to 0.2)	0.9148
LDL cholesterol, mmol/L	2.6 (0.9)	2.6 (0.7)	2.7 (0.9)	2.6 (0.7)	0 (−0.2 to 0.1)	0.7215	0 (−0.1 to 0.2)	0.7278
Triglycerides, mmol/L	1.3 (0.6)	1.3 (0.6)	1.3 (0.6)	1.3 (0.8)	0 (−0.2 to 0.2)	0.9012	−0.1 (−0.3 to 0.1)	0.5403
Systolic blood pressure, mmHg	117.1 (11)	117.1 (12.7)	115.7 (12)	115.3 (12.3)	0 (−2.4 to 2.4)	0.9833	0.9 (−1.9 to 3.6)	0.5352
Diastolic blood pressure, mmHg	79.4 (6.9)	78.9 (8.4)	78.8 (8.4)	78.3 (8.6)	0.3 (−1.4 to 2)	0.7017	0.5 (−1.4 to 2.4)	0.6013

1 Values represent means (SDs), unless otherwise noted.

2 Mixed-effect models with fixed effects for randomized group, baseline levels of the respective outcome, gender, and random effects for the intercept and slope.

3 *n* = 56 for body fat percentage and non-HDL cholesterol, and *n* = 53 for LDL cholesterol.

4 *n* = 56 for non-HDL cholesterol, and *n* = 54 for LDL cholesterol.

5 *n* = 57 for total meat, and *n* = 53 for LDL cholesterol.

6 *n* = 50 for LDL cholesterol.

7 *n* = 113 for body fat percentage, *n* = 112 for non-HDL cholesterol, and *n* = 107 for LDL cholesterol.

8 *n* = 113 for total meat, and *n* = 103 for LDL cholesterol.

9 Primary outcome.

10 Not a prespecified outcome measure.

In a sensitivity analysis using BOCF, analyses suggested that the estimated intervention impact on total meat consumption from the main analysis was robust to this imputation technique at both follow-ups (**Supplemental Table 1**). There was no evidence that the intervention meaningfully changed the nutritional composition of participants’ diets relative to the control condition (**Table 3**).

**TABLE 3:** Nutritional outcomes^1^

	Baseline		4 weeks		8 weeks		Adjusted mean difference from baseline (95% CI)			
	Intervention (*n* = 58)	Control (*n* = 57)^2^	Intervention (*n* = 57)	Control (*n* = 57)^2^	Intervention (*n* = 57)	Control (*n* = 56)^3^	4 weeks (*n* = 114)^4^	*P* value	8 weeks (*n* = 113)^5^	*P* value
Energy, kJ	8020 (1729)	8099 (1857)	7681 (1775)	7852 (1884)	7463 (1441)	7805 (1936)	—		—	
Energy, kcal	1917 (413)	1936 (444)	1836 (424)	1877 (451)	1784 (345)	1865 (463)	−25.9 (−123 to 71)	0.6005	−64 (−165 to 37)	0.2121
Total fat	72 (24)	73 (25)	66 (19)	71 (24)	66 (20)	72 (25)	−4.8 (−10 to 1)	0.0952	−5.4 (−11 to 1)	0.0844
Saturated fat	23 (10)	24 (9)	21 (8)	24 (9)	20 (8)	26 (15)	−2.4 (−5 to 0)	0.0361	−4.2 (−8 to 0)	0.0368
Monounsaturated fat	9 (6)	9 (6)	8 (6)	8 (5)	7 (4)	9 (6)	−1.2 (−3 to 1)	0.2409	−2.7 (−4 to −1)	0.0026
Polyunsaturated fat	4 (3)	5 (4)	4 (4)	4 (3)	4 (2)	5 (3)	0.2 (−1 to 1)	0.7689	−0.7 (−2 to 0)	0.1749
Fiber	20 (8)	21 (8)	22 (8)	20 (8)	20 (7)	20 (8)	2.2 (0–4)	0.0394	−0.1 (−2 to 2)	0.9408
Protein	82 (24)	82 (24)	72 (17)	76 (23)	73 (20)	78 (22)	−3.7 (−9 to 1)	0.1432	−3.9 (−9 to 1)	0.1557
Total carbohydrate	209 (55)	215 (54)	210 (53)	210 (47)	204 (46)	206 (55)	3.7 (−8 to 16)	0.5341	2 (−11 to 15)	0.7614
Sugar	67 (27)	74 (29)	66 (26)	72 (26)	58 (25)	71 (33)	−1.5 (−7 to 4)	0.6141	−7.1 (−14 to 0)	0.0526
Sodium	1908 (933)	1965 (811)	1934 (856)	1861 (762)	1791 (682)	1864 (577)	98.2 (−158 to 354)	0.4522	−40.8 (−241 to 159)	0.6893
Vitamin A	599 (550)	748 (1014)	795 (988)	751 (689)	759 (635)	626 (552)	76.5 (−221 to 374)	0.6146	175.6 (−32 to 384)	0.098
Vitamin C	95 (95)	110 (88)	95 (96)	101 (88)	78 (53)	99 (88)	1.2 (−27 to 29)	0.9343	−14.9 (−38 to 8)	0.2075
Iron	5 (4)	6 (4)	5 (3)	6 (3)	5 (4)	6 (3)	−0.6 (−1 to 0)	0.2014	−0.55 (−2 to 1)	0.4007
Calcium	483 (306)	470 (291)	492 (283)	494 (322)	455 (321)	417 (272)	−5.6 (−106 to 95)	0.9128	28.8 (−77 to 134)	0.5925
Potassium	961 (531)	1160 (635)	896 (523)	1080 (570)	846 (381)	1017 (596)	−88.8 (−258 to 81)	0.3042	−68.7 (−231 to 93)	0.406

1 Values represent means (SDs) of 7-day food diaries recorded using the MyFitnessPal app.

2 *n* = 56 for saturated fat, monounsaturated fat, polyunsaturated fat, vitamin A, vitamin C, iron, calcium, and potassium.

3 *n* = 55 for saturated fat, monounsaturated fat, polyunsaturated fat, vitamin A, vitamin C, iron, calcium, and potassium.

4 *n* = 113 for saturated fat, monounsaturated fat, polyunsaturated fat, vitamin A, vitamin C, iron, calcium, and potassium.

5 *n* = 112 for saturated fat, monounsaturated fat, polyunsaturated fat, vitamin A, vitamin C, iron, calcium, and potassium.

The intervention increased intentions, positive attitudes, perceived control, and subjective norms of eating a low-meat diet and of using meat substitutes, and decreased participants’ attachment to meat (**Table 4**). The proportion of participants adopting a meat-reduced or meat-free eating identity in the intervention group was 27% (compared with 9% in the control group) at 4 weeks and 45% (compared with 11% in the control group) at 8 weeks (Table 4). There was no evidence that the intervention significantly changed participants’ desire for meat substitutes to resemble meat (Table 4).

**TABLE 4:** Psychosocial variables related to meat consumption and use of meat substitutes

	Baseline		4 weeks		8 weeks		Adjusted mean difference from baseline (95% CI)			
	Intervention (*n* = 58)	Control (*n* = 57)	Intervention (*n* = 57)	Control (*n* = 56)	Intervention (*n* = 56)	Control (*n* = 56)	4 weeks (*n* = 113)	*P* value	8 weeks (*n* = 112)	*P* value
Intentions to eat a low-meat diet	4 (1.4)	4.3 (1.3)	5.4 (1.5)	4.1 (1.3)	5.7 (1.3)	4 (1.5)	1.1 (0.7–1.5)	<0.0001	1.5 (1.1–1.9)	<0.0001
Attitudes towards eating a low-meat diet	5.1 (1.2)	5.4 (1)	6 (1.3)	5.2 (1.1)	6.2 (1)	5.1 (1.3)	0.5 (0.2–0.9)	0.0016	0.8 (0.4–1.1)	<0.0001
Perceived control to eat a low-meat diet	4.8 (1.1)	5.2 (1.1)	5.5 (1.2)	4.8 (1.1)	5.6 (1.3)	4.8 (1.2)	0.5 (0.07–0.8)	0.0209	0.6 (0.2–1)	0.0037
Subjective social norms of eating a low-meat diet	4 (1.4)	3.9 (1.5)	4.3 (1.3)	3.8 (1.4)	4.5 (1.4)	3.9 (1.5)	0.5 (0.2–0.9)	0.0025	0.7 (0.4–1)	<0.0001
Attachment to meat	4.4 (0.9)	4.4 (0.7)	4 (0.9)	4.4 (0.9)	3.9 (1)	4.3 (0.9)	−0.4 (−0.2 to −0.6)	<0.0001	−0.4 (−0.2 to −0.6)	0.0006
Desire for meat substitutes to be as similar as possible to meat	3.5 (1.6)	4.2 (1.8)	4 (1.7)	3.4 (1.8)	3.8 (1.7)	3.6 (1.9)	0.2 (−0.3 to 0.7)	0.4277	−0.2 (−0.6 to 0.3)	0.546
Intentions to use meat substitutes	4 (−1.3)	4.2 (1.2)	5.5 (1.3)	3.6 (1.4)	5.5 (1.4)	4 (1.5)	1.7 (1.3–2.1)	<0.0001	1.3 (0.9–1.7)	<0.0001
Attitudes towards using meat substitutes	4.9 (−1.1)	5 (1)	5.7 (1.1)	4.8 (1)	5.7 (1.1)	4.8 (1)	0.9 (0.6–1.2)	<0.0001	0.8 (0.5–1.1)	<0.0001
Perceived control to use meat substitutes	4.9 (−1)	5.2 (1)	5.5 (1.1)	4.7 (1.1)	5.3 (1.3)	4.8 (1.1)	0.6 (0.3–1)	0.0004	0.4 (0.03–0.8)	0.0335
Subjective social norms of using meat substitutes	3.7 (−1.5)	3.7 (1.4)	4 (1.4)	3.4 (1.3)	4.1 (1.6)	3.6 (1.5)	0.5 (0.2–0.8)	0.0004	0.4 (0.2–0.7)	0.0025
Eating identity, *n* (%)	—	—	—	—	—	—	5.1 (1.4–18.8)	0.014	9.7 (3–31.1)	0.0001
Meat-eating identity	53 (93)	53 (91)	42 (91)	51 (74)	31 (55)	50 (89)	—		—	
Meat-reducing identity	4 (7)	5 (9)	14 (9)	5 (25)	24 (43)	6 (11)	—		—	
Non-meat-eating identity	0 (0)	0 (0)	1 (0)	0 (2)	1 (2)	0 (0)	—		—	

The intervention led to a significant reduction in body weight of −0.6 kg (95% CI: −1.2 to −0.1) at 8 weeks; however, there was no evidence that the intervention significantly altered body composition, blood pressure, or lipid fractions (Table 2).

Our modelling provided early evidence that the intervention significantly reduced the GHG emissions (in CO_2_-eq) and the land requirements of recipients’ diets at both follow-ups (**Table 5**).

**TABLE 5:** Greenhouse gas and land use estimates

	Baseline		4 weeks		8 weeks					
	Mean (SD)		Mean (SD)		Mean (SD)		Adjusted mean difference from baseline (95% CI)			
	Intervention (*n* 58)	Control (*n* 57)	Intervention (*n* 57)	Control (*n* 57)	Intervention (*n* 57)	Control (*n* 56)	4 weeks	*P* value	8 weeks	*P* value
Greenhouse gas emissions from diet, kg of CO2-equivalent per week	39.6 (15.7)	42.7 (16)	28.6 (11)	40.1 (13.9)	31.7 (13.2)	40.3 (16.3)	−10.5 (−14.8 to −6.3)	<0.0001	−7.5 (−12.6 to −2.5)	0.0032
Land use from diet, m2 per week	116.4 (71.6)	129.1 (70.7)	78.3(49.9)	120.9 (66.2)	88.5 (58.8)	115.6 (70.1)	−39.4 (−59.9 to −18.9)	0.0002	−23.6 (−46 to −1.3)	0.0382

## Discussion

An intervention offering meat substitutes and behavioral support to promote meat reduction halved meat consumption in 4 weeks and led to a smaller sustained reduction at 4 weeks after the intervention completion, with proportional increases in the consumption frequency of meat substitutes. There was no evidence that the intervention changed the consumption of other principal food groups or the nutritional composition of participants’ diets in a clinically meaningful way. The intervention increased intentions, positive attitudes, subjective norms, and perceived control to eat a low-meat diet and use meat substitutes; increased the likelihood of taking up a meat-reducing or non-meat-eating identity; and reduced attachment to meat during and after its delivery. The intervention led to small but significant weight loss, but there was no evidence of an effect on blood pressure, blood lipid profiles, or body composition. Finally, the intervention led to statistically significant reductions in the estimated GHG emissions and land use from participants’ diets.

This was the first RCT assessing the behavioral, nutritional, psychosocial, health, and environmental impacts of an intervention aiming to reduce meat consumption through replacement with meat substitutes. Measuring outcomes during and after the intervention completion provided some early evidence on the sustained impact of the intervention. The use of food diaries allowed participants to record their eating behavior prospectively, thus reducing the error associated with retrospective dietary measures. The diaries assessed the frequency and portion size of meat consumption and identified the sub-types of meat, recognizing the large variation in their health and environmental impacts (16). Measuring meat consumption over 7 days provided a better estimation of habitual meat intake compared to studies assessing meat consumption over fewer days. Blinding the researcher conducting the study visits to the randomization sequence and blinding the researchers coding food diaries and entering data in the database to the group allocation reduced the risk of bias. The analysis of the environmental impact of recipients’ diets was based on empirically collected data rather than modelling how diets *might* change following the introduction of the intervention.

Our study has some limitations. The multicomponent nature of the intervention means that it was impossible to quantitatively assess which intervention component(s) causally influenced the outcomes. Many of the meat substitutes were frozen or suitable for freezing, so it is possible that the sustained intervention effect at 8 weeks reflects eating up free supplies rather than new purchases, and may therefore overestimate the sustained impact on meat and meat substitute consumption. Participants were only recruited among adult-only households close to Oxford, were highly educated, and had a greater proportion of females compared to the British population. By design, we only recruited participants with high meat intakes, and the intervention effect is likely to be lower, in absolute terms, in people who consume less meat. Participants were not blind to the group allocation and may have felt greater motivation to respond to the intervention given it was administered by a researcher, increasing its apparent effect. The food diaries themselves might have influenced participants’ meat consumption, but they were used by participants in both study groups. Estimates of food consumption may have been affected by recording errors: indeed, the energy intakes for both study groups and at all measurement points suggested underreporting, which might have underestimated the intervention effect in absolute terms (19). The manual disaggregation of meat from food diaries may have introduced some nondifferential error in the measurement of the primary outcome. Some psychosocial outcomes included in this study were not measured with validated scales, but the items employed were co-designed with members of the public and were seen as clear, comprehensible, and conveying the intended meaning. The analysis of the environmental impact of replacing meat with alternative plant-based products (RE-MAP) was included post hoc as an exploratory analysis. Environmental impact data were not available for every food group included in the analysis, and we occasionally used similar foods as proxies. We were not able to account for the individual production methods, and therefore used average category values. Disaggregated data (**Supplemental Table 2**) were only available for meat, and the environmental impacts of other food groups had to be estimated based on measured consumption frequencies and assumed portion sizes. The analysis focusing on GHG emissions was based on an aggregate measure of GHGs (i.e., CO_2_-eq). This metric does not fully capture the different long-term effects of different GHGs, and future research should model the expected long-term climate change impact of replacing meat with meat substitutes, differentiating between different GHGs. Despite its limitations, CO_2_-eq is still used in many environmental impact analyses, and may provide a useful interim indication of the impact of replacing meat with meat substitutes.

### Results in context of other studies

Two previous pre-post studies showed that providing meat substitutes for free as part of a wider behavioral intervention was associated with reduced meat consumption during and after the intervention (20, 21). Our RCT supports a causal role of this intervention approach.

Previously published systematic reviews found that providing information on the health and environmental benefits of eating less meat led to greater intentions to consume a low-meat diet but did not lead to actual changes in meat consumption (22). This suggests that the information leaflets alone in RE-MAP are unlikely to have halved meat consumption, but may have acted synergistically with the meat alternatives, boosting individuals’ motivation to experiment with (unfamiliar) meat substitutes. Systematic reviews of intervention studies did not identify prior evidence of the effectiveness of recipes and success stories on meat consumption. However, these intervention components are in line with general principles from behavioral sciences, suggesting that people's capability to perform a behavior and the perception that other people are successfully engaging in a behavior can influence behavior (23, 24).

Previous observational evidence suggested that the desire for meat substitutes to be similar to meat is lower among people who frequently consume meat substitutes (4). This led us to hypothesize that encouraging frequent consumption of meat substitutes through an intervention might decrease recipients’ desire for meat substitutes to be similar to meat and, over time, encourage intervention recipients to shift towards a more “traditional” plant-based diet, which might have greater health and environmental benefits. However, in our trial, people's desire for similarity between meat and meat substitutes remained stable or increased as a result of the intervention. Encouraging intervention recipients to transition from meat substitutes to a more traditional plant-based diet might therefore require additional active interventions.

Concern has been expressed that meat alternatives may contain more salt than equivalent meat products (25). Salt intake is difficult to measure accurately using food diaries because of salt added during cooking or at the table, but we found no evidence of clinically meaningful differences in intakes of sodium or other nutrients as a result of the intervention. Diets high in meat have been associated with an increased risk of coronary heart disease (26). Here, the intervention led to a reduction in weight, but we found no other changes in cardiovascular risk factors, perhaps because of the short duration of exposure. In line with our findings, a recent RCT found that replacing meat for plant-based substitutes reduced body weight (27). The same study also found beneficial effects on LDL cholesterol after 8 weeks of replacing meat with meat substitutes, which we did not observe in the present study. This discrepancy might be due to the shorter duration of our intervention period or the fact that our behavioral study did not require intervention participants to exclude meat from their diets or control participants to consume meat.

Overall, the results of RE-MAP suggest that harnessing the mere exposure effect from providing meat substitutes is an effective way to reduce meat consumption and that people using meat substitutes, who are motivated to reduce their consumption, swap meat for substitutes without changing other aspects of their diets. The costs of the intervention mean that RE-MAP is unlikely to be a scalable option in its current form, but it shows that increasing exposure to and consumption of meat substitutes can lead to important reductions in meat intake, including after the intervention. Other interventions that might work through similar mechanisms to achieve population-level impacts include repositioning meat substitute products in more prominent areas of grocery stores, providing vouchers for meat substitutes or free samples, or incentivizing customers to replace meat with meat substitutes when shopping online. Although these results are promising, previous studies suggest that a sustained 50% or greater reduction in ruminant red meat is required to keep food systems within safe planetary boundaries, and it is likely that greater use of meat substitutes will need to be accompanied by additional interventions to reduce meat consumption to achieve the scale of change that is needed for sustainable food systems (28, 29).

## Supplementary Material

nqab414_Supplemental_Tables

## Data Availability

Data described in the manuscript will be made available upon request pending application and approval.

## Notes

This study was funded by the Wellcome Trust, Our Planet Our Health program [Livestock, Environment, and People (LEAP), award number 205212/Z/16/Z]. FB's time on this project was funded by the Medical Research Council, Green Templeton College, and the NIHR School for Primary Care Research. NMA, PA and SAJ are supported by the NIHR Oxford Biomedical Research Centre. PA and SAJ are also supported by the NIHR Applied Research Collaboration and are NIHR senior investigators.

Supplemental Tables 1 and 2 are available from the “Supplementary data” link in the online posting of the article and from the same link in the online table of contents at https://academic.oup.com/ajcn/.

Abbreviations used: BOCF, baseline observation carried forward; GHG, greenhouse gas; RCT, randomized controlled trial; RE-MAP, replacing meat with alternative plant-based products.
