# Supplementary material for: Replacing meat with alternative plant-based products (RE-MAP): a randomized controlled trial of a multicomponent behavioral intervention to reduce meat consumption
Source: Am J Clin Nutr. 2021 Dec 27;115(5):1357–66. doi: 10.1093/ajcn/nqab414 (PMC9071457; doi:10.1093/ajcn/nqab414)
Supplement: nqab414_Supplemental_Tables [file nqab414_supplemental_tables.zip › Supplementary Table 1- Disaggregated meat consumption.pptx]

## Slide 1
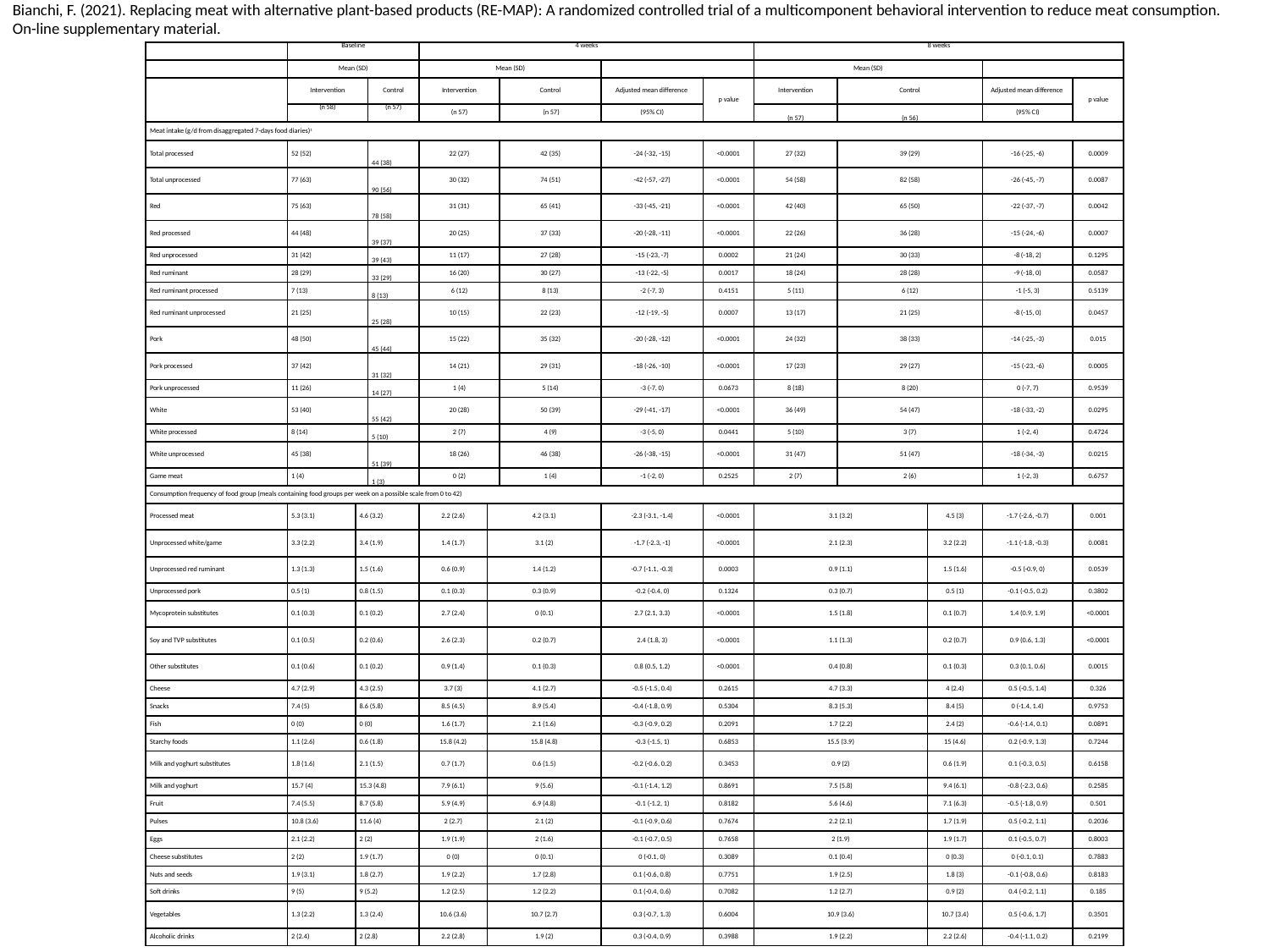

Bianchi, F. (2021). Replacing meat with alternative plant-based products (RE-MAP): A randomized controlled trial of a multicomponent behavioral intervention to reduce meat consumption.
On-line supplementary material.
| | Baseline | | | 4 weeks | | | | | 8 weeks | | | | |
| --- | --- | --- | --- | --- | --- | --- | --- | --- | --- | --- | --- | --- | --- |
| | Mean (SD) | | | Mean (SD) | | | | | Mean (SD) | | | | |
| | Intervention | | Control | Intervention | | Control | Adjusted mean difference | p value | Intervention | Control | | Adjusted mean difference | p value |
| | (n 58) | | (n 57) | (n 57) | | (n 57) | (95% CI) | | (n 57) | (n 56) | | (95% CI) | |
| Meat intake (g/d from disaggregated 7-days food diaries)1 | | | | | | | | | | | | | |
| Total processed | 52 (52) | | 44 (38) | 22 (27) | | 42 (35) | -24 (-32, -15) | <0.0001 | 27 (32) | 39 (29) | | -16 (-25, -6) | 0.0009 |
| Total unprocessed | 77 (63) | | 90 (56) | 30 (32) | | 74 (51) | -42 (-57, -27) | <0.0001 | 54 (58) | 82 (58) | | -26 (-45, -7) | 0.0087 |
| Red | 75 (63) | | 78 (58) | 31 (31) | | 65 (41) | -33 (-45, -21) | <0.0001 | 42 (40) | 65 (50) | | -22 (-37, -7) | 0.0042 |
| Red processed | 44 (48) | | 39 (37) | 20 (25) | | 37 (33) | -20 (-28, -11) | <0.0001 | 22 (26) | 36 (28) | | -15 (-24, -6) | 0.0007 |
| Red unprocessed | 31 (42) | | 39 (43) | 11 (17) | | 27 (28) | -15 (-23, -7) | 0.0002 | 21 (24) | 30 (33) | | -8 (-18, 2) | 0.1295 |
| Red ruminant | 28 (29) | | 33 (29) | 16 (20) | | 30 (27) | -13 (-22, -5) | 0.0017 | 18 (24) | 28 (28) | | -9 (-18, 0) | 0.0587 |
| Red ruminant processed | 7 (13) | | 8 (13) | 6 (12) | | 8 (13) | -2 (-7, 3) | 0.4151 | 5 (11) | 6 (12) | | -1 (-5, 3) | 0.5139 |
| Red ruminant unprocessed | 21 (25) | | 25 (28) | 10 (15) | | 22 (23) | -12 (-19, -5) | 0.0007 | 13 (17) | 21 (25) | | -8 (-15, 0) | 0.0457 |
| Pork | 48 (50) | | 45 (44) | 15 (22) | | 35 (32) | -20 (-28, -12) | <0.0001 | 24 (32) | 38 (33) | | -14 (-25, -3) | 0.015 |
| Pork processed | 37 (42) | | 31 (32) | 14 (21) | | 29 (31) | -18 (-26, -10) | <0.0001 | 17 (23) | 29 (27) | | -15 (-23, -6) | 0.0005 |
| Pork unprocessed | 11 (26) | | 14 (27) | 1 (4) | | 5 (14) | -3 (-7, 0) | 0.0673 | 8 (18) | 8 (20) | | 0 (-7, 7) | 0.9539 |
| White | 53 (40) | | 55 (42) | 20 (28) | | 50 (39) | -29 (-41, -17) | <0.0001 | 36 (49) | 54 (47) | | -18 (-33, -2) | 0.0295 |
| White processed | 8 (14) | | 5 (10) | 2 (7) | | 4 (9) | -3 (-5, 0) | 0.0441 | 5 (10) | 3 (7) | | 1 (-2, 4) | 0.4724 |
| White unprocessed | 45 (38) | | 51 (39) | 18 (26) | | 46 (38) | -26 (-38, -15) | <0.0001 | 31 (47) | 51 (47) | | -18 (-34, -3) | 0.0215 |
| Game meat | 1 (4) | | 1 (3) | 0 (2) | | 1 (4) | -1 (-2, 0) | 0.2525 | 2 (7) | 2 (6) | | 1 (-2, 3) | 0.6757 |
| Consumption frequency of food group (meals containing food groups per week on a possible scale from 0 to 42) | | | | | | | | | | | | | |
| Processed meat | 5.3 (3.1) | 4.6 (3.2) | | 2.2 (2.6) | 4.2 (3.1) | | -2.3 (-3.1, -1.4) | <0.0001 | 3.1 (3.2) | | 4.5 (3) | -1.7 (-2.6, -0.7) | 0.001 |
| Unprocessed white/game | 3.3 (2.2) | 3.4 (1.9) | | 1.4 (1.7) | 3.1 (2) | | -1.7 (-2.3, -1) | <0.0001 | 2.1 (2.3) | | 3.2 (2.2) | -1.1 (-1.8, -0.3) | 0.0081 |
| Unprocessed red ruminant | 1.3 (1.3) | 1.5 (1.6) | | 0.6 (0.9) | 1.4 (1.2) | | -0.7 (-1.1, -0.3) | 0.0003 | 0.9 (1.1) | | 1.5 (1.6) | -0.5 (-0.9, 0) | 0.0539 |
| Unprocessed pork | 0.5 (1) | 0.8 (1.5) | | 0.1 (0.3) | 0.3 (0.9) | | -0.2 (-0.4, 0) | 0.1324 | 0.3 (0.7) | | 0.5 (1) | -0.1 (-0.5, 0.2) | 0.3802 |
| Mycoprotein substitutes | 0.1 (0.3) | 0.1 (0.2) | | 2.7 (2.4) | 0 (0.1) | | 2.7 (2.1, 3.3) | <0.0001 | 1.5 (1.8) | | 0.1 (0.7) | 1.4 (0.9, 1.9) | <0.0001 |
| Soy and TVP substitutes | 0.1 (0.5) | 0.2 (0.6) | | 2.6 (2.3) | 0.2 (0.7) | | 2.4 (1.8, 3) | <0.0001 | 1.1 (1.3) | | 0.2 (0.7) | 0.9 (0.6, 1.3) | <0.0001 |
| Other substitutes | 0.1 (0.6) | 0.1 (0.2) | | 0.9 (1.4) | 0.1 (0.3) | | 0.8 (0.5, 1.2) | <0.0001 | 0.4 (0.8) | | 0.1 (0.3) | 0.3 (0.1, 0.6) | 0.0015 |
| Cheese | 4.7 (2.9) | 4.3 (2.5) | | 3.7 (3) | 4.1 (2.7) | | -0.5 (-1.5, 0.4) | 0.2615 | 4.7 (3.3) | | 4 (2.4) | 0.5 (-0.5, 1.4) | 0.326 |
| Snacks | 7.4 (5) | 8.6 (5.8) | | 8.5 (4.5) | 8.9 (5.4) | | -0.4 (-1.8, 0.9) | 0.5304 | 8.3 (5.3) | | 8.4 (5) | 0 (-1.4, 1.4) | 0.9753 |
| Fish | 0 (0) | 0 (0) | | 1.6 (1.7) | 2.1 (1.6) | | -0.3 (-0.9, 0.2) | 0.2091 | 1.7 (2.2) | | 2.4 (2) | -0.6 (-1.4, 0.1) | 0.0891 |
| Starchy foods | 1.1 (2.6) | 0.6 (1.8) | | 15.8 (4.2) | 15.8 (4.8) | | -0.3 (-1.5, 1) | 0.6853 | 15.5 (3.9) | | 15 (4.6) | 0.2 (-0.9, 1.3) | 0.7244 |
| Milk and yoghurt substitutes | 1.8 (1.6) | 2.1 (1.5) | | 0.7 (1.7) | 0.6 (1.5) | | -0.2 (-0.6, 0.2) | 0.3453 | 0.9 (2) | | 0.6 (1.9) | 0.1 (-0.3, 0.5) | 0.6158 |
| Milk and yoghurt | 15.7 (4) | 15.3 (4.8) | | 7.9 (6.1) | 9 (5.6) | | -0.1 (-1.4, 1.2) | 0.8691 | 7.5 (5.8) | | 9.4 (6.1) | -0.8 (-2.3, 0.6) | 0.2585 |
| Fruit | 7.4 (5.5) | 8.7 (5.8) | | 5.9 (4.9) | 6.9 (4.8) | | -0.1 (-1.2, 1) | 0.8182 | 5.6 (4.6) | | 7.1 (6.3) | -0.5 (-1.8, 0.9) | 0.501 |
| Pulses | 10.8 (3.6) | 11.6 (4) | | 2 (2.7) | 2.1 (2) | | -0.1 (-0.9, 0.6) | 0.7674 | 2.2 (2.1) | | 1.7 (1.9) | 0.5 (-0.2, 1.1) | 0.2036 |
| Eggs | 2.1 (2.2) | 2 (2) | | 1.9 (1.9) | 2 (1.6) | | -0.1 (-0.7, 0.5) | 0.7658 | 2 (1.9) | | 1.9 (1.7) | 0.1 (-0.5, 0.7) | 0.8003 |
| Cheese substitutes | 2 (2) | 1.9 (1.7) | | 0 (0) | 0 (0.1) | | 0 (-0.1, 0) | 0.3089 | 0.1 (0.4) | | 0 (0.3) | 0 (-0.1, 0.1) | 0.7883 |
| Nuts and seeds | 1.9 (3.1) | 1.8 (2.7) | | 1.9 (2.2) | 1.7 (2.8) | | 0.1 (-0.6, 0.8) | 0.7751 | 1.9 (2.5) | | 1.8 (3) | -0.1 (-0.8, 0.6) | 0.8183 |
| Soft drinks | 9 (5) | 9 (5.2) | | 1.2 (2.5) | 1.2 (2.2) | | 0.1 (-0.4, 0.6) | 0.7082 | 1.2 (2.7) | | 0.9 (2) | 0.4 (-0.2, 1.1) | 0.185 |
| Vegetables | 1.3 (2.2) | 1.3 (2.4) | | 10.6 (3.6) | 10.7 (2.7) | | 0.3 (-0.7, 1.3) | 0.6004 | 10.9 (3.6) | | 10.7 (3.4) | 0.5 (-0.6, 1.7) | 0.3501 |
| Alcoholic drinks | 2 (2.4) | 2 (2.8) | | 2.2 (2.8) | 1.9 (2) | | 0.3 (-0.4, 0.9) | 0.3988 | 1.9 (2.2) | | 2.2 (2.6) | -0.4 (-1.1, 0.2) | 0.2199 |
