# Supplementary material for: Replacing meat with alternative plant-based products (RE-MAP): a randomized controlled trial of a multicomponent behavioral intervention to reduce meat consumption
Source: Am J Clin Nutr. 2021 Dec 27;115(5):1357–66. doi: 10.1093/ajcn/nqab414 (PMC9071457; doi:10.1093/ajcn/nqab414)
Supplement: nqab414_Supplemental_Tables [file nqab414_supplemental_tables.zip › Supplementary Table 2- Sensitivity analysis.pptx]

## Slide 1
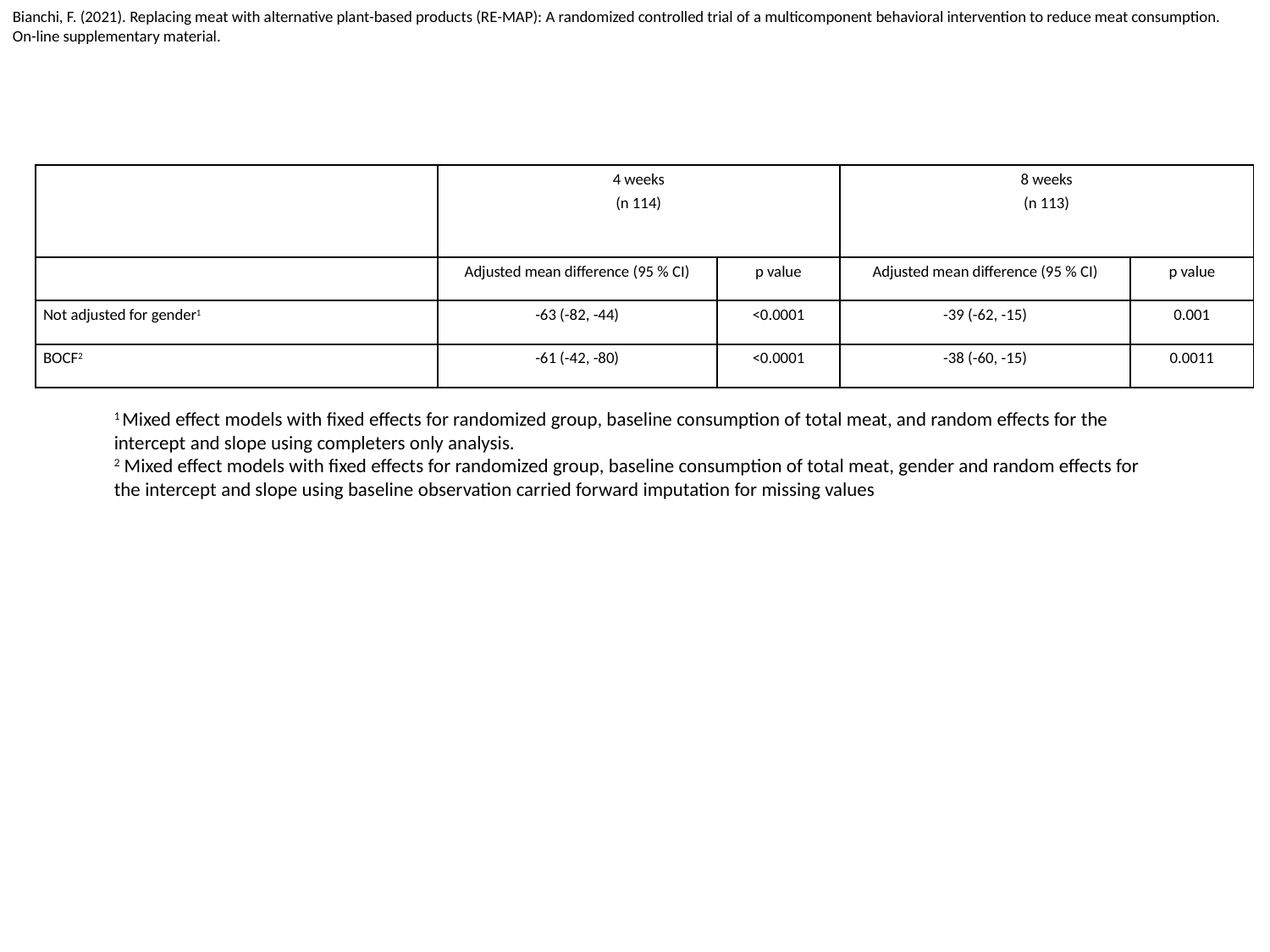

Bianchi, F. (2021). Replacing meat with alternative plant-based products (RE-MAP): A randomized controlled trial of a multicomponent behavioral intervention to reduce meat consumption.
On-line supplementary material.
| | 4 weeks (n 114) | | 8 weeks (n 113) | |
| --- | --- | --- | --- | --- |
| | Adjusted mean difference (95 % CI) | p value | Adjusted mean difference (95 % CI) | p value |
| Not adjusted for gender1 | -63 (-82, -44) | <0.0001 | -39 (-62, -15) | 0.001 |
| BOCF2 | -61 (-42, -80) | <0.0001 | -38 (-60, -15) | 0.0011 |
1 Mixed effect models with fixed effects for randomized group, baseline consumption of total meat, and random effects for the intercept and slope using completers only analysis.
2 Mixed effect models with fixed effects for randomized group, baseline consumption of total meat, gender and random effects for the intercept and slope using baseline observation carried forward imputation for missing values
